# Probiotics Reduce Vaginal Candidiasis in Pregnant Women via Modulating Abundance of *Candida* and *Lactobacillus* in Vaginal and Cervicovaginal Regions

**DOI:** 10.3390/microorganisms10020285

**Published:** 2022-01-26

**Authors:** Xin Yee Ang, Uma Mageswary Mageswaran, Yi Li Fiona Chung, Boon Kiat Lee, Siti Nur Afiqah Azhar, Nurhanis Syazni Roslan, Ili Farhana Binti Saufian, Nor Sheila Mustaffa, Ermadina Mohamad Kalam, Aini Farhah Ibrahim, Normala Abdul Wahid, Zakuan Zainy Deris, Chern-Ein Oon, Wan Fadhlina Wan Adnan, Salina Sany, Min-Tze Liong

**Affiliations:** 1 School of Industrial Technology, Universiti Sains Malaysia, Penang 11800, Malaysia; xinyee0919@outlook.com (X.Y.A.); umamageswary1901@gmail.com (U.M.M.); fionacyl92@gmail.com (Y.L.F.C.); boonkiatlee92@gmail.com (B.K.L.); 2 School of Medical Sciences, Universiti Sains Malaysia, Kelantan 16150, Malaysia; afeeqahazhar@gmail.com (S.N.A.A.); syazniroslan1234@yahoo.com (N.S.R.); zakuanzainy@outlook.com (Z.Z.D.); fadhlinaadnan@outlook.com (W.F.W.A.); 3 Kota Bharu Health Clinic, Ministry of Health Malaysia, Kelantan 15200, Malaysia; llifarhana@yahoo.com; 4 Hospital Universiti Sains Malaysia (HUSM), Health Campus, Kelantan 16150, Malaysia; 5 Kubang Kerian Health Clinic, Ministry of Health Malaysia, Kelantan 16150, Malaysia; sheila.mustafa@yahoo.com; 6 Pengkalan Chepa Health Clinic, Ministry of Health Malaysia, Kelantan 16100, Malaysia; ermadina_kalam@yahoo.com; 7 Wakaf Che Yeh Health Clinic, Ministry of Health Malaysia, Kelantan 15100, Malaysia; norainiahmad99@yahoo.com; 8 Sejahtera Centre, Universiti Sains Malaysia, Penang 11800, Malaysia; normalawahid@outlook.com; 9 Institute for Research in Molecular Medicine (INFORMM), Universiti Sains Malaysia, Penang 11800, Malaysia; chern.oon@usm.my; 10 Advanced Medical and Dental Institute, Universiti Sains Malaysia, Penang 13200, Malaysia

**Keywords:** probiotic, vaginal candidiasis, pregnant, *Candida*, *Lactobacillus*, inflammation

## Abstract

We previously reported on the effects of a lactobacilli probiotic (SynForU-HerCare; two capsules/day of 9.5 log CFU/capsule) in improving symptoms of vaginal irritation, discharge and burning in pregnant women with vaginal candidiasis upon administration for 8 weeks, accompanied by improved emotional and social quality of life parameters. Thus, the present study aimed to analyse vaginal microbiota and inflammatory changes in hope to better understand the improved clinical symptoms as observed previously. Patients in the probiotic group showed a decreased abundance of *Candida glabrata* after 8 weeks (*p* = 0.009) in the lower vaginal region, while patients in the placebo group did not show any changes over time. In the higher vaginal and cervicovaginal regions, patients in the placebo group showed a decreased abundance of *Candida albicans* only within 4 weeks (*p* < 0.05) but no changes in abundance of *C. glabrata* over time, while patients in the probiotic group showed a continuous decreased abundance of *C. albicans* and *C. glabrata* over 8 weeks (*p* < 0.05). Patients in the placebo group also had a decreased abundance of *Lactobacillus crispatus* over 4 weeks (*p* = 0.023) in the lower vaginal region and a decreased abundance of *L. jensenii* over 8 weeks in the cervicovaginal region (*p* = 0.001). Meanwhile, patients in the probiotic group had an increased abundance of *L. crispatus* in the lower vaginal region after 8 weeks (*p* = 0.012) and *Lactobacillus jensenii* over 4 weeks in the cervicovaginal region (*p* < 0.001). Inflammation may have occurred in both low and high vaginal regions, predominantly observed by the increased concentration of pro-inflammatory cytokine TNF-alpha in patients from the placebo group (*p* < 0.05), while the administration of probiotics has shortened the period of inflammation as observed from the reduced need for anti-inflammatory cytokine IL-4 and IL-10 over time (*p* < 0.05). Taken together, our present new data further support previous findings that probiotic SynForU-HerCare had a beneficial effect against vaginal candidiasis in pregnant women via modulation of the vaginal microbiota and microenvironment.

## 1. Introduction

Vulvovaginal infections are the most common gynaecological illnesses among women, with recurrences that are defined as more than three episodes per year, affecting nearly 8% of women globally [1]. The three common vulvovaginal infections are bacterial vaginosis, vulvovaginal candidiasis and trichomoniasis. Changes in the vulvovaginal regions as affected by these infections often create a niche for the pathogenesis of other infections, leading to mixed infections and co-infections, which when left untreated, will not only affect the female reproductive health, but may also result in many foster infections/diseases and adverse pregnancy outcomes [2]. Vulvovaginal candidiasis (VVC) is the most prevalent human candida infection resulting in inflammation of the vulva or vagina and affecting 75% of reproductive-age women. The majority of VVC is caused by *Candida albicans* and *Candida glabrata*, which are opportunistic fungi that invade the mucous membrane of the vagina, leading to exuberant mucosal inflammatory responses [3]. This eventually leads to symptoms of vaginal itchiness, irritation, swelling, pain during sexual intercourse, soreness, discomfort, redness and vaginal discharge [4].

VVC is also the most common form of fungal infection in pregnant mothers, which may cause systemic infections in neonate and has been associated with low birth weight and premature delivery [5]. Pregnancy increases the frequency of vaginal *Candida* colonization and it is thought to be the consequences of increased levels of circulating estrogens and deposition of glycogen and other substrates in the vagina during pregnancy [6]. Azole-based antifungal drugs remain as the top choice to treat VVC including the pregnant population. However, this has caused various concerns attributed to the emergence of drug-resistant yeasts, while prolonged exposure to fluconazole, as in cases of recurrences, can shift the predominant vaginal yeast flora from *Candida albicans* to more intrinsically azole-resistant species such as *Candida krusei* or *Candida glabrata*, typically in immunosuppressed women, and may be exacerbated in healthy women by use of over-the-counter products for self-treatment of VVC. Cross-resistance between OTC drugs (miconazole, clotrimazole, and tioconazole) and fluconazole has been observed in clinical isolates of *C. glabrata* and *C. albicans* [7,8].

Probiotics are live microorganisms that exert health benefits to the host upon consumption in sufficient amounts [9]. Although probiotics have been used commonly for gut health [10], increasing evidence over the years has illustrated the benefits of probiotics beyond that of gut maintenance [11,12]. Probiotics have been documented to maintain and modulate microbiota profiles in the gut and vagina, and they are able to inhibit pathogenic *Candida* species. These have given rise to the concept of using probiotics for the treatment of VVC. We have previously reported the use of a multi-species lactobacilli probiotic (SynForU-HerCare) to reduce the recurrences of vaginal candidiasis (VC) in pregnant women. SynForU-HerCare is a probiotic product containing dairy-isolated lactobacilli, namely *Lactiplantibacillus plantarum* (former *Lactobacillus plantarum*) LP115, *Lactobacillus helveticus* LA25 (previously identified as *L. acidophilus* [13,14]), *Lacticaseibacillus rhamnosus* (former *Lactobacillus rhamnosus*) LRH10, *Lacticaseibacillus paracasei* (former *Lactobacillus paracasei*) LPC12, *Limosilactobacillus fermentum* (former *Lactobacillus fermentum*) LF26, and *Lactobacillus delbrueckii* subsp. *Lactis* LDL114. These strains of lactobacilli were developed primarily for women’s health with patents in Taiwan (2013) and China (2018), specifically for their ability to adhere to HeLa cells (a human cervical carcinoma cell line) and to produce hydrogen peroxide, which inhibits infectious urogenital bacteria in women such as *Salmonella, Escherichia coli*, *C. albicans*, and *Gardnerella vaginalis* [13,14]. Our previous randomized, double-blind and placebo-controlled study involving 78 pregnant women with VC showed that the oral administration of SynForU-HerCare for 8 weeks reduced VVC symptoms and recurrences of VC, accompanied by improved emotional and social distress attributed to VC as compared to the placebo, indicating that probiotics could be a potential strategy for the maintenance of vaginal health during pregnancy [8].

This study is a continuation of our previous work, where we continue to evaluate the effects of SynForU-HerCare against the abundance of *Candida* and *Lactobacillus* in the high- and low-vaginal and cervicovaginal regions of pregnant women with VC, accompanied by assessments for inflammatory responses in these regions. We hypothesize that the recurrences of VC in pregnant women are attributed to a disruption of vaginal lactobacilli, leading to overgrowth of vaginal *Candida*, while probiotics were able to restore such a disruption.

## 2. Materials and Methods

### 2.1. Study Design and Population

The study design population has been described previously [8]. Briefly, this was a randomized, double-blinded, placebo-controlled study, enrolling pregnant women at 14–32 weeks of pregnancy and confirmed with VC by an obstetrics and gynaecology specialist and/or a medical doctor. Subjects (n = 78) were allocated 1:1 to receiving probiotic tablets (9.5 log CFU/capsule of total lactobacilli) or an indistinguishable placebo tablet (containing maltodextrin carrier only). All patients were treated for VC via insertion of vaginal suppository Candid V3 (containing 200 mg of clotrimazole per tablet) for three days. Intervention consisted of orally administered two capsules/day of lactobacilli or placebo for 8 weeks. The study was approved by the JEPeM-USM Review Panel on Clinical Studies (Approval number USM/JEPeM/18090421), and by the Medical Research and Ethics Committee (MREC) Ministry of Health Malaysia (approval number NMRR-19-2591-50662) and was registered at ClinicalTrials.gov (identifier number NCT03940612). 

### 2.2. Vaginal Swabs and Cervicovaginal Lavages

Vaginal swabs and cervicovaginal lavages were collected by our team of obstetrics and gynaecology specialist in the sequence of low vaginal swab (LVS), followed by high vaginal swab (HVS) and finally cervicovaginal lavage (CVL) at baseline (week 0), week 4 and week 8. All samples were collected without any contact with urine and external parts of the reproductive system using speculum and commercial sterile nylon flocked swabs. LVS samples were collected via inserting the swab into the vagina past the labia to distance of about 3 cm, while HVS samples were collected from the ventral fornix. All swabs were placed in sterile saline (3 mL). CVL samples were collected via instilling 10 mL of sterile saline to wash the ectocervical and vaginal mucosal surfaces and then reaspirated from the posterior fornix into a sterile collection tube. All samples were kept at −80 °C until further analyses. 

### 2.3. Abundance of Candida and Lactobacillus Species

DNA was extracted from all samples using the TIANamp Genomic DNA kit, following the manufacturer’s instructions (Tiangen, Beijing, China) for quantitative PCR (qPCR) analysis of *Candida/Lactobacillus* abundance. Species-specific primers used to determine abundance of *Candida/Lactobacillus* are listed in Table 1. The total qPCR volume was 20 µL, with 1 µL of extracted DNA, 100–400 nM forward/reverse primers, UV-treated RNase-free H_2_O, and 2× Fast SYBR green PCR master mix- CAPITAL qPCR Green Mix (biotechrabbit, Berlin, Germany) was used. qPCR was carried out using a Step-One Plus real-time PCR machine (Agilent, Santa Clara, CA, USA) with the following thermal conditions: an activation step of 95 °C for 3 min followed by 40 cycles of 95 °C for 10 s to denature and annealing at 60 °C for 30 s. Standard curves constructed from serially diluted DNA of *C. albicans* AB2, *C. glabrata* L-3329, *L. jensenii* 8586 and *L. crispatus* C25 were used to extrapolate *Candida* and *Lactobacillus* colony-forming unit (CFU) per millilitre, respectively.

### 2.4. Inflammatory Proteins

LVS and HVS samples were analysed for the concentrations of interleukin-4 and -10, tumour necrosis factor (TNF)-α and interferon (IFN)-γ using enzyme-linked immunosorbent assay (ELISA) kits (Sunlong Biotech, Hangzhou, China) following the manufacturer’s instructions. Concentrations of inflammatory proteins were standardized upon total protein content as determined using the Bradford assay [18].

### 2.5. Statistical Analyses

Data were analysed using SPSS version 24.0 (SPSS Inc., Chicago, IL, USA). The primary hypothesis of this study involved differential efficacy between the two treatment groups of probiotics and placebo. Considering the skewed distribution and non-parametric nature of our data, differences of scale data between probiotic and placebo groups or between two different time points, were compared using the Mann–Whitney U test, while nominal data were compared using the Chi-square test. All tests were two-sided with *p* < 0.05 as considered statistically significant, and data are presented as mean value ± standard error unless stated otherwise. 

## 3. Results

### 3.1. Baseline Characteristics

A total of 78 patients were recruited for this study as previously described. At baseline (week 0), one sample from the probiotic group had a low concentration of DNA where detection of abundance for *Candida/Lactobacillus* were unable to be performed, yielding 38 samples for analyses. A similar problem persisted at week 4, with two samples from the placebo group and one sample from the probiotic group, yielding 37 and 38 samples for analyses, respectively. Meanwhile, two samples from the placebo group at week 8 had low concentration of DNA. Additionally, a total of 12 patients from the placebo group and 8 patients from the probiotic group went into labour before sample collection and/or failure to obtain sample due to travel restrictions attributed to national lockdown for COVID-19, yielding 25 and 31 samples for analyses, respectively. Insignificant differences were observed in the general characteristics of probiotic and placebo patients despite a reduction in sample size (Table 2). 

### 3.2. Abundance of Candida

Patients in the placebo group did not show any changes in abundance of *Candida albicans* and *Candida glabrata* over 8 weeks in the LVS region amid treatment using vaginal suppository drug clotrimazole (Table 3). Patients in the probiotic group also did not show any changes in abundance of *Candida albicans* over 8 weeks but showed a decreased abundance of *Candida glabrata* after 8 weeks (*p* = 0.009). This showed that probiotics prevented the re-occurrence of *Candida glabrata* and also inhibited growth over a prolonged period in the LVS region.

Patients in the placebo group showed a decreased in abundance of *Candida albicans* after 4 weeks in the HVS region (*p* = 0.004), most probably attributed to the use of vaginal suppository drug clotrimazole. However, this effect was not observed at week 8. Patients in the probiotic group showed a decrease in abundance of *Candida albicans* after 4 weeks and 8 weeks as compared to baseline (*p* = 0.005), indicating a prevention of candidiasis recurrences over prolonged period. Meanwhile, the placebo group did not show any changes in abundance of *Candida glabrata* throughout 8 weeks. However, patients consuming probiotics showed a continuous decrease in abundance of *Candida glabrata* over 8 weeks (*p* = 0.046), indicating not just a prevention but also inhibitory effects against *Candida glabrata*.

Patients in the placebo group showed a decreased in abundance of *Candida albicans* after 4 weeks in the cervicovaginal region (*p* < 0.001) but failed to sustain a low abundance over a prolonged period, as shown in the increased abundance between week 4 and week 8 (*p* < 0.001). Meanwhile, patients consuming probiotics showed a decrease in abundance of *Candida albicans* after 8 weeks (*p* = 0.010) indicating potential benefits over a prolonged period. The placebo group also showed a continuous decrease in abundance of *Candida glabrata* after 4 weeks and thereafter (*p* = 0.001), while patients in the probiotic group only showed a decrease after 8 weeks (*p* = 0.008).

### 3.3. Abundance of Lactobacillus

Insignificant differences were observed for abundance of *L. jensenii* in both treatment groups over 8 weeks in the LVS region (Table 4). Patients in the placebo group showed a decrease in abundance of *L. crispatus* after 4 weeks (*p* = 0.023) which was maintained at this low concentration thereafter. Patients in the probiotic group showed an increase in abundance of *L. crispatus* after 8 weeks (*p* = 0.012), indicating that the administration of probiotics increased the population of *L. crispatus* in the LVS region. Meanwhile, insignificant differences were observed in changes of lactobacilli in the HVS region between treatment groups. 

Patients in the placebo group showed a continuous decrease in abundance of *L. jensenii* over 8 weeks in the cervicovaginal region (*p* = 0.001). Meanwhile, patients in the probiotic group showed an increase in abundance of *L. jensenii* over 4 weeks (*p* < 0.001) but decreased thereafter (*p* < 0.001). Insignificant differences were observed in changes of *L. crispatus* in the cervicovaginal region between treatment groups. 

### 3.4. Inflammatory Proteins

Concentration of proinflammatory cytokine IFN-gamma was reduced in the placebo group only after week 8 (*p* < 0.001) but was decreased since week 4 and thereafter in the probiotic group (*p* = 0.011) in the LVS region (Table 5). Proinflammatory cytokine TNF-alpha increased after week 4 in both groups but continued to increase thereafter until week 8 in the placebo group (*p* < 0.001), while remaining unchanged in the probiotic group for the same period of time. Anti-inflammatory cytokines IL-4 and IL-10 increased within week 4 in the probiotic group (*p* < 0.001) and decreased thereafter (*p* < 0.001), while the placebo group remained unchanged within week 4 and decreased after week 8 (*p* < 0.001).

Insignificant changes were observed in the concentration of proinflammatory cytokine IFN-gamma over time between treatment groups in the HVS region. The concentration of proinflammatory cytokine TNF-alpha in the placebo group increased after week 4 (*p* < 0.001) and remained until week 8 (*p* = 0.181), while a decrease was observed in the probiotic group after week 4 (*p* = 0.018). The concentration of anti-inflammatory IL-4 decreased in the placebo group after week 4 (*p* < 0.001) but increased thereafter (*p* = 0.026), while a continuous decrease was observed in the probiotic group over 8 weeks (*p* = 0.001). The concentration of anti-inflammatory IL-10 continuously increased in the placebo group over 8 weeks (*p* < 0.001) but only increased after 4 weeks in the probiotic group and maintained thereafter.

## 4. Discussion

While VVC is one of the most common vulvovaginal infections in women, a higher prevalence is detected in pregnant women with increased risks attributed to recurrent episodes of infections. Symptomatic recurrences among pregnant women are also highest during the second and third trimester during pregnancy, mainly attributed to decreased cell-mediated immunity, increased estrogen levels and increased vaginal mucosal glycogen production that facilitates adherence of yeast to vaginal mucosal epithelial cells during these phases of pregnancy [19]. Evidence has suggested that VVC during pregnancy is associated with an increased risk of premature rupture of membranes and poor pregnancy outcome, while eradication of VVC in pregnancy via the use of clotrimazole reduces the risk of preterm birth. Although VVC-induced chorioamnionitis is rare, several cases have been reported of intraamniotic infection caused by *C. albicans* and *C. glabrata*, leading to preterm rupture of membranes or preterm labour, where such a progression could lead to foetal fatality [20]. While treatment using clotrimazole has rare and few side effects, a small number of patients (<10%) have reported vulvar or vaginal burning sensation, rash, hives, blisters, burning, itching, peeling, redness, swelling, pain, or other signs of skin irritation. It is thus hoped that a natural dietary intervention such as that of using probiotics may alleviate these side effects of treatment, albeit rare, and prevent recurrences among pregnant women that ultimately may reduce pregnancy and birth complications.

Our present study shows that a probiotic consisting of a lactobacilli mixture in conjunction with clotrimazole prevented recurrences of *C. glabrata* after 8 weeks in the lower vaginal region, although a similar effect was not observed for *C. albicans*. *C. glabrata* is the second most common *Candida* species in humans and is a lesser pathogenic commensal of the vagina as compared to *C. albicans.* This may be the reason that the inhibitory effect of lactobacilli was more prominent against *C. glabrata* than *C. albicans.* While clotrimazole was developed as a vaginal insert treatment that primarily targets *C. albicans,* recent clinical isolates have identified increasing resistance of *C. glabrata* against the drug [21]. Our current data shows an alternative of using lactobacilli against *C. glabrata* in the effort to possibly reduce dependency on clotrimazole. 

Meanwhile, in the higher vaginal region, clotrimazole reduced the abundance of *C. albicans* after 4 weeks, but such a reduction was not sustained over a prolonged period of time as seen in the placebo group, while a reduction was also observed at week 8 in the probiotic group. Patients on the placebo showed a consistent abundance of both *Candida* species over time in the lower vaginal region but decreased abundance of *C. albicans* after 4 weeks in the higher vaginal region, while it increased in both *C. albicans* and *C. glabrata* in the cervicovaginal region between week 4 and week 8. While *C. albicans* and *C. glabrata* are facultative anaerobes, they proliferate better under aerobic conditions [22]. We postulate that clotrimazole was sufficient in preventing the overgrowth of these *Candida* in the lower vaginal region, but insufficient to inhibit and prevent recurrences. The higher vaginal region would be more anaerobic than the lower vaginal regions. *C. albicans* is prone to produce hyphae under anaerobicity which increases its survival, virulence and pathogenicity [23]. While *C. glabrata* is unable to produce hyphae; it is prone to attach to the hyphae of *C. albicans*, leading to increased survival, virulence and pathogenicity as well. This may explain the unchanged abundance of *C. glabrata* over time in higher vaginal regions despite treatment using clotrimazole in the placebo patients. The administration of probiotics reduced the abundance of both *C. albicans* and *C. glabrata* continuously over 8 weeks in the higher vaginal region, indicating not just growth inhibition against *Candida* but also the prevention of recurrences typically in the higher vaginal regions, an area of stubborn virulence and pathogenicity.

The reduced abundance of *C. glabrata* in the lower vaginal region upon administration of probiotics also occurred in a microenvironment with increased abundance of *L. crispatus*, while the reduced abundance of *Candida* in higher vaginal regions upon administration of probiotics had an increased abundance of *L. jensenii* instead. *L. crispatus* and *L. jensenii* are the main lactobacilli species among a healthy vaginal microbiota, where a reduced abundance is often observed in vaginitis patients such as bacterial vaginosis and VVC [24]. Predominant vaginal LABs are also grouped into community state types (CST) grouped as I, II, III, IV, V, where each is dominated by *L. crispatus*, *L. gasseri*, *L. iners*, polymicrobial *Lactobacillus* and bacterial vaginosis-associated bacteria, and *L. jensenii*, respectively. *L. crispatus*- and *L. jensenii*-dominated vaginal microbiota form CST I and V, respectively, an indication of a healthy vaginal microenvironment. However, the influence of hormones particularly oestradiol during pregnancy can stimulate the transition of CST I (*L. crispatus*-dominated) to other CSTs including those of transitional and diseases states [25]. Based on these, our present study shows that the administration of probiotics shifted the vaginal microenvironment towards a healthier state, where lower vaginal regions were dominated by *L. crispatus* (CST I) and higher vaginal regions dominated by *L. jensenii* (CST V), amid the presence of vaginal candidiasis. 

VVC is often accompanied by inflammation, primarily caused by the ability of *Candida*, typically *C. albicans*, to co-regulate with the expression of genes encoding for virulence factors such as secreted aspartyl proteases and candidalysin, causing tissue damages. While vaginal epithelial cells counter *Candida* invasion via epithelial shedding, secretion of mucin and strong interepithelial cell connections, an increased fungal burden often overcomes the tolerance threshold, triggering an intense inflammatory response [26]. In our present study, inflammation may have occurred in both low and high vaginal regions, predominantly observed by the increased concentrations of pro-inflammatory cytokine TNF-alpha. The administration of probiotics has shortened the period of inflammation in low vaginal regions, as observed from the increased pro-inflammatory cytokines IL-4 and IL-10 after 4 weeks in the probiotic group, while placebo did not show any changes over the same period but attributed to increased pro-inflammatory cytokine TNF-alpha over 8 weeks in the placebo group but only increased over 4 weeks in the probiotic group which decreased thereafter. This was also in line with the unchanged or increased abundance of *Candida* in both low and high vaginal regions over time in the placebo patients, but reduced abundance of *Candida* in patients consuming probiotics. Probiotics have been associated with inhibitory activities against a myriad of human pathogens [27] and anti-inflammatory properties due to the production of inflammation-inhibitive metabolites [28].

## 5. Conclusions

While our previous report showed that the probiotic SynForU-HerCare improved clinical symptoms in pregnant women with vaginal candidiasis, our present study further strengthened these observations by providing evidence on modulation of vaginal microbiota typical abundance of *Candida* and lactobacilli and inflammatory responses, typically concentrations of TNF-alpha, IL-4 and IL-10. All patients received treatment of vaginal insert clotrimazole. Our present study showed that such a treatment was insufficient to prevent recurrences of *Candida* growth over time, while the addition of probiotics aided in preventing such recurrences. Amid concerns of increased drug resistance among *Candida*, our current data also shows an alternative of using lactobacilli in the effort to possibly reduce dependency on candidiasis drugs. Taken altogether, our data suggest that probiotics can be a crucial alternative against vaginal candidiasis in pregnant women via inhibition of *Candida*, prevention of recurrences and reduction of vaginal inflammation. 

## Figures and Tables

**Table 1:** Sequences of species-specific primers used to determine abundance of *Candida* and *Lactobacillus* species.

Microorganism		Sequence (5′-3′)	Reference
*C. albicans*	*C. albicans*-F	ATTGCTTGCGGCGGTAACGTCC	[15]
	*Candida* spp-R	TCTTTTCCTCCGCTTATTGATATGC	
*C. glabrata*	*C. glabrata*-F	TAGGTTTTACCAACTCGGTGTT	
	*Candida* spp-R	TCTTTTCCTCCGCTTATTGATATGC	
*L. crispatus*	*L. crispatus*-F	AGCGAGCGGAACTAACAGATTTAC	[16]
	*L. crispatus*-R	AGCTGATCATGCGATCTGCTT	
*L. jensenii*	*L. jensenii*-F	AAGTCGAGCGAGCTTGCCTATAGA	[17]
	*L. jensenii*-R	CTTCTTTCATGCGAAAGTAGC	

**Table 2:** Baseline characteristics of patients with vaginal candidiasis randomly assigned to 8 weeks of double-blind treatment with either placebo or lactobacilli at reduced sample sizes due to a low concentration of DNA and/or failure of sample collections.

	Week 0			Week 4			Week 8		
Baseline Characteristics	Placebo	Probiotic	*p* Value	Placebo	Probiotic	*p* Value	Placebo	Probiotic	*p* Value
Sample size (n)	39	38		37	38		25	31	
Trimester period	23.56 ± 0.96	23.76 ± 0.88	0.996	27.38 ± 0.99	27.71 ± 0.89	0.890	31.40 ± 1.06	30.71 ± 0.92	0.614
Age	28.38 ± 0.82	28.89 ± 0.72	0.553	28.41 ± 0.82	28.87 ± 0.71	0.580	28.20 ± 1.04	29.16 ± 0.74	0.257
Body weight (kg)	62.10 ± 1.81	66.55 ± 2.65	0.239	62.38 ± 1.90	66.79 ± 2.61	0.259	62.08 ± 2.47	65.45 ± 3.07	0.531
Height (cm)	155.32 ± 1.12	156.59 ± 0.96	0.635	155.09 ± 1.16	156.30 ± 0.96	0.663	154.06 ± 1.46	155.98 ± 1.10	0.557
BMI	25.89 ± 0.88	26.99 ± 0.93	0.227	26.08 ± 0.91	27.17 ± 0.90	0.233	26.26 ± 1.16	26.75 ± 1.09	0.510

*p* value obtained via Mann–Whitney U test. All data are presented as mean value ± standard error.

**Table 3:** Relative abundance of *Candida albicans* and *Candida glabrata* in low vaginal, high vaginal and cervicovaginal regions from patients with vaginal candidiasis randomly assigned to 8 weeks of double-blind treatment with either placebo or lactobacilli.

Sample Type	Abundance (Log CFU/mL qPCR)	Placebo						Probiotic					
		Weeks			*p* Value			Weeks			*p* Value		
		WO	W4	W8	W0 vs. W4	W0 vs. W8	W4 vs. W8	WO	W4	W8	W0 vs. W4	W0 vs. W8	W4 vs. W8
Low vaginal swab (LVS)	*Candida albicans*	4.451 ± 0.108	4.331 ± 0.104	4.343 ± 0.137	0.556	0.357	0.779	4.480 ± 0.148	4.351 ± 0.167	4.332 ± 0.153	0.341	0.458	0.787
	*Candida glabrata*	2.045 ± 0.093	1.987 ± 0.096	2.041 ± 0.115	0.384	0.770	0.327	1.918 ± 0.055	1.932 ± 0.069	1.666 ± 0.072	0.485	0.009 *	0.006 *
High vaginal swab (HVS)	*Candida albicans*	4.076 ± 0.129	3.615 ± 0.167	3.688 ± 0.222	0.004 *	0.148	0.451	4.691 ± 0.162	4.112± 0.179	3.973 ± 0.184	0.011 *	0.005 *	0.691
	*Candida glabrata*	1.688 ± 0.113	1.419 ± 0.146	1.416 ± 0.189	0.082	0.070	0.818	1.689 ± 0.094	1.340± 0.108	1.499 ± 0.123	0.010 *	0.046 *	0.462
Cervico vaginal lavage (CVL)	*Candida albicans*	4.615 ± 0.083	3.678 ± 0.116	4.207 ± 0.100	<0.001 *	0.001 *	<0.001 *	4.375 ± 0.110	4.361 ± 0.106	3.816 ± 0.180	0.446	0.010 *	0.003 *
	*Candida glabrata*	2.009 ± 0.096	1.472 ± 0.125	1.821 ± 0.158	0.001 *	0.309	0.035 *	1.823 ± 0.733	1.916 ± 0.086	1.559 ± 0.110	0.328	0.008 *	0.005 *

* *p* < 0.050. LVS and CVL samples were standardized per 5 ng/ul total DNA, while HVS samples were standardized per 10 ng/ul total DNA. All data are presented as mean value ± standard error.

**Table 4:** Relative abundance of *Lactobacillus jensenii* and *Lactobacillus crispatus* in low vaginal, high vaginal and cervicovaginal regions from patients with vaginal candidiasis randomly assigned to 8 weeks of double-blind treatment with either placebo or lactobacilli.

Sample Type	Abundance (Log CFU/mL qPCR)	Placebo						Probiotic					
		Weeks			*p* Value			Weeks			*p* Value		
		WO	W4	W8	W0 vs. W4	W0 vs. W8	W4 vs. W8	WO	W4	W8	W0 vs. W4	W0 vs. W8	W4 vs. W8
Low vaginal swab (LVS)	*Lactobacillus jensenii*	4.038 ± 0.180	4.196 ± 0.208	3.998 ± 0.229	0.644	0.624	0.976	3.566 ± 0.166	3.987 ± 0.187	4.297 ± 0.203	0.826	0.059	0.158
	*Lactobacillus crispatus*	4.384 ± 0.207	3.715 ± 0.334	4.228 ± 0.308	0.023 *	0.319	0.110	3.673 ± 0.300	4.161 ± 0.315	4.343 ± 0.298	0.111	0.012 *	0.407
High vaginal swab (HVS)	*Lactobacillus jensenii*	4.127 ± 0.174	4.492 ± 0.225	4.273 ± 0.198	0.382	0.553	0.943	3.893 ± 0.126	3.963 ± 0.178	3.973 ± 0.184	0.826	0.059	0.158
	*Lactobacillus crispatus*	4.603 ± 0.219	4.749 ± 0.191	4.785 ± 0.276	0.433	0.372	0.809	5.117 ± 0.215	4.834 ± 0.224	4.811 ± 0.214	0.310	0.467	0.504
Cervico vaginal lavage (CVL)	*Lactobacillus jensenii*	4.180 ± 0.210	3.774 ± 0.214	3.209 ± 0.292	0.026 *	0.001 *	0.020 *	3.205 ± 0.181	4.108 ± 0.133	3.305 ± 0.257	<0.001 *	0.572	<0.001 *
	*Lactobacillus crispatus*	3.450 ± 0.355	3.978 ± 0.295	4.140 ± 0.353	0.173	0.124	0.668	4.174 ± 0.304	4.130 ± 0.281	4.451 ± 0.277	0.937	0.095	0.098

* *p* < 0.050. LVS and CVL samples were standardized per 5 ng/uL total DNA, while HVS samples were standardized per 10 ng/uL total DNA. All data are presented as mean value ± standard error.

**Table 5:** Concentrations of inflammatory proteins in low and high vaginal regions from patients with vaginal candidiasis randomly assigned to 8 weeks of double-blind treatment with either placebo or lactobacilli.

Sample Type	Inflammatory Proteins	Placebo						Probiotic					
		Weeks			*p* Value			Weeks			*p* Value		
		WO	W4	W8	W0 vs. W4	W0 vs. W8	W4 vs. W8	WO	W4	W8	W0 vs. W4	W0 vs. W8	W4 vs. W8
Low vaginal swab (LVS)	IFN-gamma (pg/ug protein)	2.057 ± 0.216	2.113 ± 0.159	1.021 ± 0.091	0.394	<0.000 *	<0.000 *	2.730 ± 0.169	2.132 ± 0.127	2.026 ± 0.205	0.011 *	0.004 *	0.321
	TNF-alpha (ng/ug protein)	0.706 ± 0.070	1.234 ± 0.228	1.428 ± 0.177	0.007 *	<0.000 *	<0.000 *	0.746 ± 0.115	2.440 ± 0.476	1.249 ± 0.052	<0.000 *	<0.000 *	0.217
	IL-4 (pg/ug protein)	0.932 ± 0.053	0.913 ± 0.049	0.390 ± 0.022	0.779	<0.000 *	<0.000 *	0.622 ± 0.328	1.171 ± 0.058	0.402 ± 0.020	<0.000 *	<0.000 *	<0.000 *
	IL-10 (pg/ug protein)	0.612 ± 0.080	0.581 ± 0.099	0.227 ± 0.024	0.934	<0.000 *	<0.000 *	0.403 ± 0.025	1.642 ± 0.361	0.203 ± 0.019	<0.000 *	<0.000 *	<0.000 *
High vaginal swab (HVS)	IFN-gamma (pg/ug protein)	3.137 ± 0.209	3.747 ± 0.237	1.261 ± 0.075	0.071	<0.000 *	<0.000 *	3.839 ± 0.233	3.732 ± 0.164	1.230 ± 0.068	0.988	<0.000 *	<0.000 *
	TNF-alpha (ng/ug protein)	0.779 ± 0.066	1.572 ± 0.115	2.836 ± 1.152	<0.000 *	<0.000 *	0.181	0.577 ± 0.039	2.003 ± 0.125	1.678 ± 0.118	<0.000 *	<0.000 *	0.018 *
	IL-4 (pg/ug protein)	1.357 ± 0.095	0.420 ± 0.049	0.483 ± 0.044	<0.000 *	<0.000 *	0.026 *	1.180 ± 0.068	0.629 ± 0.032	0.488 ± 0.053	<0.000 *	<0.000 *	0.001 *
	IL-10 (pg/ug protein)	0.867 ± 0.051	0.905 ± 0.149	1.600 ± 0.143	0.028 *	<0.000 *	<0.000 *	0.588 ± 0.043	0.836 ± 0.061	0.964 ± 0.104	0.001 *	<0.000 *	0.745

* *p* < 0.050. All samples were standardized per total protein. All data are presented as mean value ± standard error.

## References

[1] Willems H.M.E., Ahmed S.S., Liu J., Xu Z., Peters B.M. (2020). Vulvovaginal Candidiasis: A Current Understanding and Burning Questions. J. Fungi.

[2] Kalia N., Singh J., Kaur M. (2019). Immunopathology of Recurrent Vulvovaginal Infections: New Aspects and Research Directions. Front Immunol..

[3] Jeanmonod R., Jeanmonod D. (2020). Vaginal Candidiasis (Vulvovaginal Candidiasis). StatPearls [Internet].

[4] Okonkwo N., Umeanaeto P., Okonkwo N., Umeanaeto P. (2020). Prevalence of Vaginal Candidiasis among Pregnant Women in Nnewi Town of Anambra State, Nigeria: A Recent Perspective. Theory and Applications of Microbiology and Biotechnology.

[5] Rasti S., Asadi M.A., Taghriri A., Behrashi M., Mousavie G. (2014). Vaginal Candidiasis Complications on Pregnant Women. Jundishapur J. Microbiol..

[6] Sobel J.D. (2007). Vulvovaginal candidosis. Lancet Lond. Engl..

[7] Mathema B., Cross E., Dun E., Park S., Bedell J., Slade B., Williams M., Riley L., Chaturvedi V., Perlin D.S. (2001). Prevalence of Vaginal Colonization by Drug-Resistant *Candida* Species in College-Age Women with Previous Exposure to Over-the-Counter Azole Antifungals. Clin. Infect. Dis..

[8] Ang X.-Y., Chung F.-Y.-L., Lee B.-K., Azhar S.N.A., Sany S., Roslan N.S., Ahmad N., Yusof S.M., Abdullah N., Rahman N.N.N.A. (2021). Lactobacilli Reduce Recurrences of Vaginal Candidiasis in Pregnant Women: A Randomized, Double-Blind, Placebo-Controlled Study. J. Appl. Microbiol..

[9] Nutrition Division (2006). Probiotics in Food: Health and Nutritional Properties and Guidelines for Evaluation—Report of a Joint FAO/WHO Expert Consultation on Evaluation of Health and Nutritional Properties of Probiotics in Food Including Powder Milk with Live Lactic Acid Bacteria [Internet].

[10] Hor Y.-Y., Lew L.-C., Lau A.S.-Y., Ong J.-S., Chuah L.-O., Lee Y.-Y., Choi S., Rashid F., Wahid N., Sun Z. (2018). Probiotic *Lactobacillus casei* Zhang (LCZ) alleviates respiratory, gastrointestinal & RBC abnormality via immuno-modulatory, anti-inflammatory & anti-oxidative actions. J. Funct. Foods..

[11] Liong M.T., Shah N.P. (2005). Acid and Bile Tolerance and Cholesterol Removal Ability of Lactobacilli Strains. J. Dairy Sci..

[12] Liu Y.-W., Liong M.-T., Tsai Y.-C. (2018). New perspectives of *Lactobacillus plantarum* as a probiotic: The gut-heart-brain axis. J. Microbiol..

[13] Chang I.J., Lin J.S., Chu M.T., Li H.F. (2013). A Novel Strain of *lactobacillus* and Its Use in Inhibition of Vaginitis.

[14] Zhang Y., Li X., Zhu M., Lin J. (2018). *Lactobacillus* for Inhibiting Pathogenic Bacteria of Vaginitis and Application Thereof.

[15] Ishida K., Yamaguchi M.U., Nakamura T.U., Dias Filho B.P., Yamada-Ogatta S.F., Nakamura C.V. (2013). Desempenho dos métodos de identificação de leveduras de água engarrafada: Alta prevalência de Candida parapsilosis. Semin. Ciênc. Biol. E Saúde.

[16] Byun R., Nadkarni M.A., Chhour K.-L., Martin F.E., Jacques N.A., Hunter N. (2004). Quantitative Analysis of Diverse *Lactobacillus* Species Present in Advanced Dental Caries. J. Clin. Microbiol..

[17] Berza N., Zodzika J., Kroica J., Reinis A., Skadins I., Zalizko P., Melngaile O., Pundure R., Lukojanova I., Vasina O. (2013). Association between *Lactobacillus* species and bacterial vaginosis-related bacteria, and bacterial vaginosis scores in small population of pregnant Latvian women. Int. J. Collab. Res. Intern. Med. Public Health.

[18] Olson B.J.S.C., Markwell J. (2007). Assays for Determination of Protein Concentration. Curr. Protoc. Protein Sci..

[19] Aguin T.J., Sobel J.D. (2015). Vulvovaginal candidiasis in pregnancy. Curr. Infect. Dis. Rep..

[20] Meizoso T., Rivera T., Fernández-Aceñero M.J., Mestre M.J., Garrido M., Garaulet C. (2008). Intrauterine candidiasis: Report of four cases. Arch Gynecol. Obstet..

[21] Costa C., Ribeiro J., Miranda I.M., Silva-Dias A., Cavalheiro M., Costa-de-Oliveira S., Rodrigues A.G., Teixeira M.C. (2016). Clotrimazole Drug Resistance in *Candida glabrata* Clinical Isolates Correlates with Increased Expression of the Drug:H+ Antiporters CgAqr1, CgTpo1_1, CgTpo3, and CgQdr2. Front. Microbiol..

[22] Biswas S.K., Chaffin W.L. (2005). Anaerobic Growth of *Candida albicans* Does Not Support Biofilm Formation Under Similar Conditions Used for Aerobic Biofilm. Curr. Microbiol..

[23] Janus M.M., Crielaard W., Volgenant C.M.C., van der Veen M.H., Brandt B.W., Krom B.P. (2017). *Candida albicans* alters the bacterial microbiome of early in vitro oral biofilms. J. Oral. Microbiol..

[24] Superti F., De Seta F. (2020). Warding Off Recurrent Yeast and Bacterial Vaginal Infections: Lactoferrin and Lactobacilli. Microorganisms.

[25] Chee W.J.Y., Chew S.Y., Than L.T.L. (2020). Vaginal microbiota and the potential of *Lactobacillus* derivatives in maintaining vaginal health. Microb. Cell Fact..

[26] Ardizzoni A., Wheeler R.T., Pericolini E. (2021). It Takes Two to Tango: How a Dysregulation of the Innate Immunity, Coupled With Candida Virulence, Triggers VVC Onset. Front. Microbiol..

[27] Tham C.S.-C., Peh K.-K., Bhat R., Liong M.-T. (2012). Probiotic properties of bifidobacteria and lactobacilli isolated from local dairy products. Ann. Microbiol..

[28] Fung W.-Y., Liong M.-T. (2010). Evaluation of proteolytic and ACE-inhibitory activity of *Lactobacillus acidophilus* in soy whey growth medium via response surface methodology. LWT.

